# Meetings of Societies

**Published:** 1928

**Authors:** 


### Meetings of Societies.

### Bristol Medico-Chirurgical Society.

*December 14th*, 1927.

DR. W. K. WILLS, President, in the Chair.

DR. D. A. ALEXANDER showed a case of a young woman with a **Tumour in the Left Hypochondrium,** present three years. He suggested it was a fibroma attached to the abdominal wall.

DR. BUSH showed a number of skiagrams, including a group of six cases showing positional abnormalities of the large intestine producing marked symptoms (in several suggestive of carcinoma), symptoms which were largely or entirely relieved by judicious abdominal massage and exercises.

DR. A. D. FRASER showed pathological specimens, including one of desmoids (*i.e.* chronic inflammatory tumours) of the abdominal wall.

MR. E. WATSON-WILLIAMS showed two specimens of **Bony Ankylosis of Malleus and Incus,** one of them exhibiting osteophytic outgrowths on both bones. The condition had not been suspected prior to the removal of the bones at operation.

DR. H. H. CARLETON read a paper on "**Problems of Hæmoptysis in Pulmonary Tuberculosis,**" reported on page 39.

MR. H. CHITTY read a paper on "**Intussusception,**" reported on page 51.

---

*January* 11*th*, 1928.

DR. W. K. WILLS, President, in the Chair.

DR. T. MILLING showed a case of **Sebaceous Cyst of the Scrotum,** with a tumour in one groin.

DR. F. G. BERGIN showed a number of skiagrams illustrating pulmonary lesions.

DR. A. E. ILES showed a woman with a **Melanoma of the Eyeball,** visible in all three classical positions ; it had produced no symptoms, and was discovered during the search for a foreign body.

DR. J. A. NIXON read a paper on "**Gastric and Duodenal Ulcer in Infants.**"

He related the case of a child eleven weeks old who had died in the Royal Infirmary of hemorrhage from a chronic gastric ulcer situated half in the stomach and half in the œsophagus. Giving a brief survey of the not inconsiderable literature on this subject, he pointed out that acute ulcers of the stomach, duodenum and even of the œsophagus were not so very rare in the new-born, but that instances of chronic ulcers were less common. In particular he referred to Cruveilhier's illustrations of ulceration of the stomach in the new-born, and quoted Landau's conclusions as to the causation of these ulcers, in which that author remarked that they were usually due to injuries of some kind during birth, especially asphyxia, and stated that almost all cases of uncomplicated *Melœna neonatorum* depended on ulcer of the stomach or duodenum. Kundrat had observed that these ulcers are occasioned by minute hemorrhages.

Dr. Nixon referred to two cases on record (Alsberg in 1920 and Palmer in 1921) where operations had been successfully performed.

The diagnosis of ulcer might be suspected in any case of melæna neonatorum unaccompanied by hemorrhage in other situations. Chronic cases were likely to be attended by marasmus, vomiting and signs of pain associated with food. If in addition to these signs hæmatemesis or bleeding from the bowel occurred the diagnosis of gastric or duodenal ulcer was almost certain.

DR. A. D. FRASER showed the specimen of the stomach from Dr. Nixon's case, and also the stomach of a child five days old which showed an acute ulcer of the duodenum.

Dr. Fraser said that the bulk of animal experiments threw no light on the subject. Recent work, however, had shown that following the injection of foreign proteins in animals gastric and duodenal ulcers were found. He thought that partial asphyxiation might provide a toxic factor in these children, and reminded the meeting that for the first three days of life every child was a potential hæmophilic.

DR. D. C. RAYNER said he was very interested in the association of this condition with asphyxia neonatorum, which was quite new to him, and asked if asphyxia had been noted in Dr. Nixon's case. In his experience the mortality of melæna neonatorum was about 60 per cent.

MR. T. CARWARDINE said that he regarded the ulceration as due indeed to a toxic factor, but by direct action on the gastric mucosa.

MR. H. J. DREW SMYTHE pointed out that there was some degree of asphyxia in all births. He did not think asphyxia explained why these very few children suffered from gastric ulcer. He had himself seen only five cases of melæna neonatorum, with no death.

DR. CAREY F. COOMBS inquired as to the occurrence of duodenal ulcer after burns in children.

DR. NIXON replied.

MR. G. R. SCARFF read a paper on "**The Incidence of Tuberculous Infection in the Faucial and Nasopharyngeal Tonsils of Children,**" a summary of which is as follows :—*

Mitchell in Edinburgh in 1916 found the incidence of tuberculous foci in tonsils removed from children for causes other than obvious tuberculous adenitis to be 9 per cent., while Weller in America in 1921 found lesions in 1·64 per cent.

To ascertain whether Mitchell's figures continued to be found in other parts of Britain at the present time a histological examination was carried out at the Middlesex Hospital of the tonsils from 100 children who attended the hospital for routine

* The full account of this work appears in the *Journal of Laryngology*, 1927.

tonsillectomy after the usual clinical examination. Of these 2 per cent. were found to show the presence of tuberculous foci.

In a further 100 cases the tonsils and the adenoid tissue were examined histologically, and in addition Dr. Whitby, bacteriologist at the hospital, carried out animal inoculation experiments in each case. In this series 2 per cent. of the tonsils and 2 per cent. of the adenoids were found to be tuberculous.

Except in one case in which the affected tonsil was much larger than the other, there was nothing in the appearance of the tonsils to be found clinically to point to tuberculous infection. All the children in both series showed enlargement of the tonsillar glands, in half the cases other glands in the anterior triangles were also enlarged. and in 11 per cent. the upper glands in the posterior triangles showed enlargement. In two of the positive cases the glandular enlargement was diagnosed clinically as tuberculous before operation, and these cases subsequently required operation for their removal. These cases emphasise the necessity for tonsillectomy in cases where tuberculous glands require to be removed, as otherwise a focus of infection remains active. The after history of the other cases showed that tonsillectomy brings about improvement where the glandular condition is early.

Inquiring into the reasons for the discrepancy in results, it was found that during the period of Mitchell's researches (war conditions) 20 per cent. of samples of milk taken in Edinburgh were positive for tubercle bacilli, 90 per cent. of the children were bottle fed, and it was the exception to boil the milk. In the present series 52 per cent. were bottle fed, and in only 6 per cent. was the milk unboiled.

The decrease can therefore be attributed in part, if not wholly, to improvement in the milk supply, and to the education of the public with regard to the danger of infected milk.

A further effect of this is to be found in the decrease in the number of patients requiring operative treatment for tuberculous adenitis—less than two-thirds the number in 1916, which must be largely due to the same cause, although not wholly comparable owing to the advance in conservative methods of treatment.

This paper was discussed by the President, Dr. Carleton, Mr. Wright, Mr. E. Watson-Williams, Mr. Adams, Mr. Claremont, Professor Nixon, Dr. Hadfield, Mr. Jackman and Dr. Alexander. Dr. P. Watson-Williams contributed a letter of welcome to Mr. Scarff, also expressing his views on the subject under discussion.

